# Design and Analysis of In-Pipe Hydro-Turbine for an Optimized Nearly Zero Energy Building

**DOI:** 10.3390/s21238154

**Published:** 2021-12-06

**Authors:** Muhammad Shahbaz Aziz, Muhammad Adil Khan, Harun Jamil, Faisal Jamil, Alexander Chursin, Do-Hyeun Kim

**Affiliations:** 1 Department of Electrical and Computer Engineering, Air University Islamabad, Islamabad 44000, Pakistan; mshahbazaziz99@gmail.com; 2 School of Automation, Central South University, Changsha 410083, China; 3 Department of Electronic Engineering, Jeju National University, Jeju-si 63243, Korea; harunjamil@hotmail.com; 4 Department of Computer Engineering (and Research Center of Advance Technology), Jeju National University, Jeju-si 63243, Korea; faisal@jejunu.ac.kr; 5 Institute of Applied Technical and Economic Research and Expert Assessment, Peoples’ Friendship University of Russia (RUDN University), Miklukho-Maklaya St., 117198 Moscow, Russia; chursin-aa@rudn.ru

**Keywords:** hybrid renewable energy system, energy management, nearly Zero Energy Building, optimization, energy efficiency, in-pipe hydro-turbine, computational fluid dynamics

## Abstract

Pakistan receives Direct Normal Irradiation (DNI) exceeding 2000 kWh/m²/annum on approximately 83% of its land, which is very suitable for photovoltaic production. This energy can be easily utilized in conjunction with other renewable energy resources to meet the energy demands and reduce the carbon footprint of the country. In this research, a hybrid renewable energy solution based on a nearly Zero Energy Building (nZEB) model is proposed for a university facility. The building in consideration has a continuous flow of water through its water delivery vertical pipelines. A horizontal-axis spherical helical turbine is designed in SolidWorks and is analyzed through a computational fluid dynamics (CFD) analysis in ANSYS Fluent 18.1 based on the K-epsilon turbulent model. Results obtained from ANSYS Fluent have shown that a 24 feet vertical channel with a water flow of 0.2309 m^3^/s and velocity of 12.66 m/s can run the designed hydroelectric turbine, delivering 168 W of mechanical power at 250 r.p.m. Based on the turbine, a hybrid renewable energy system (HRES) comprising photovoltaic and hydroelectric power is modelled and analyzed in HOMER Pro software. Among different architectures, it was found that architecture with hydroelectric and photovoltaic energy provided the best COE of $0.09418.

## 1. Introduction

The concept of nearly Zero Energy Building (nZEB) in conjunction with hybrid renewable energy systems (HRES) is expanding increasingly with time as nations begin to fully understand the importance of environmental sustainability and the role of fossil fuels in the destruction of the environment. Many hybrid renewable energy systems incorporate hydroelectric technology with other renewable energy resources subject to availability.

Renewable energy penetration was approximately 33 percent worldwide by the end of 2018 according to the International Renewable Energy Agency (IRENA). This percentage has increased from 22% to 33% in the last 17 years, which is a huge improvement but this is still is leaps and bounds behind what it should be to sustain the environment. A total of 67% of energy is obtained from fossil fuels on a global scale and this practice has pushed to the verge of environmental destruction. We have to find alternate ways to harvest clean energy from resources that are not generally considered mainstream [1].

**Figure 1:**
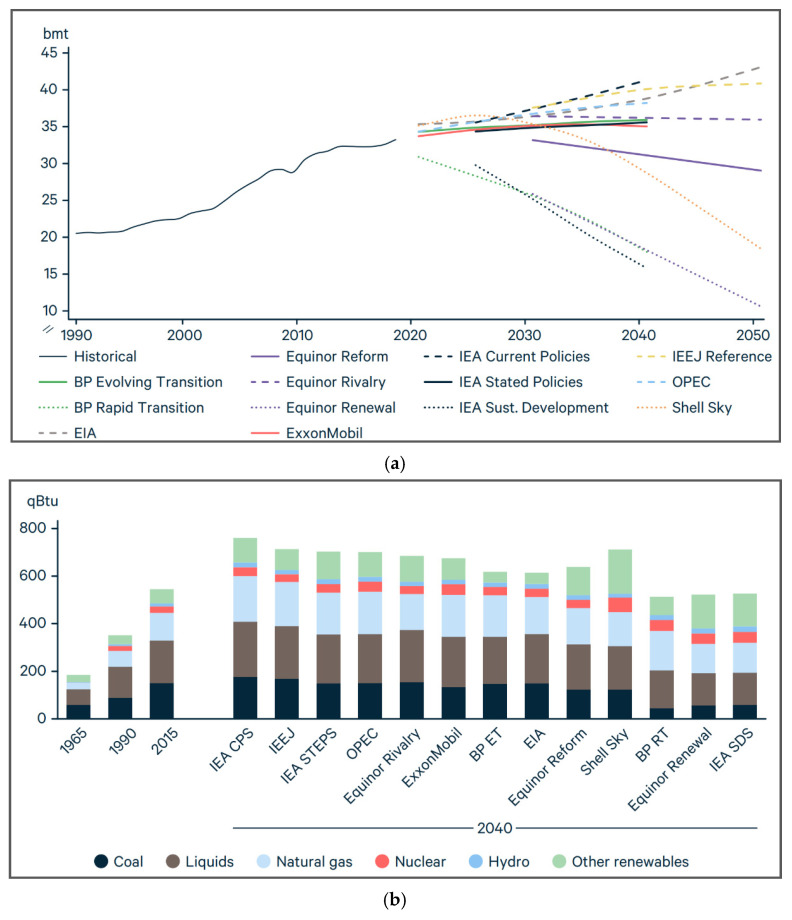
(**a**) Net global carbon dioxide emission history and predictions based on evolving energy policies around the world; (**b**) net global electricity production history and predictions based on evolving energy policies around the world [2].

Pakistan ranks 31 in terms of pollution as of mid-2020 with a pollution index of 73.48 [2]. A substantial amount of this air pollution comes from the non-renewable energy industry as 82% of electrical energy produced in Pakistan comes from non-renewable energy resources. Statistics are much worse globally and this is contributing heavily to global warming. Figure 1a,b show a comprehensive image of global carbon dioxide emission and electrical energy production distribution, respectively.

Regarding renewables, photovoltaic systems are the fastest growing technology in the world and continue to improve as we approach nanotechnology-based photovoltaic systems. According to the International Energy Agency (IEA), Solar PV generation increased by a record of 156 TWh (23%) in 2020 to 821 TWh. It demonstrated the second-largest absolute generation growth of all renewable technologies in 2020, slightly behind wind and ahead of hydropower [3].

The graphs in these figures also predict the future trajectory of renewable energy in the world under different proposed policies around the world [2].

Currently, 20% of total carbon dioxide emissions is from large commercial buildings, which are also responsible for 40% of global electricity usage [4]. Generally, these buildings are grid connected even though these buildings are capable of producing a large fraction of the electrical energy they use through the resources present within the buildings. In Pakistan, specifically, supplying electricity to these large commercial buildings in metropolitan cities results in the non-availability of electricity to distant towns and rural areas. On the other hand, environmental sustainability is also being greatly compromised as non-renewable energy resources are used to feed these power-hungry cities and it has become unfavorable to continue to use non-renewable fossil fuels to produce electricity due to environmental sustainability and socio-economic concerns. However, renewable energy technologies suffer from techno-economic barriers such as high installation costs and dependability on environmental factors (wind, sunlight). Therefore, HRES are becoming popular because of their technical and environmental benefits. HRES entail more than one renewable energy resource to attain higher reliability and a better overall economic efficiency.

The renewable energy policy of Pakistan proposes a 20% renewable energy share by 2025 and 30% by 2030 to improve sustainability and affordability [5]. The conversion of commercial buildings to nearly Zero Energy Buildings will play a significant role in achieving that goal.

Recently, the concept of nZEB has been implemented in many countries and this is seen as a good step toward reducing dependability on the local grid through self-sufficient and energy independent building construction [6]. Internationally, policies have been developed in most countries regarding new building construction and nZEB implementation. For example, Europe has decided to implement nZEB in every building constructed from 2020 under the Zero Energy Building Research Alliance (ZEBRA) 2020 program [7]. Belgium is already working on this idea and every building or a major renovation of a building must comply with nZEB standards from 2015 [6]. Korea, Japan, USA, China, Germany, France, Denmark, UK, Sweden, Australia, Singapore, and many other developed countries have similar policies that highlight the significance of nZEB in future energy systems.

Hybrid renewable energy systems have evolved significantly in recent years and many research articles have brought that evolution. Many researchers focused on HES and nZEB to develop a sustainable energy system. Seema and Bhim [8] investigated a standalone microgrid with a photovoltaic, hydroelectric, and battery system and verified that the system delivers good performance for dynamic loads. Sweeka and Ganga [9] analyzed the performance of a grid-integrated complex hybrid energy system consisting of hydropower and solar energy and concluded that the system in observation is economically feasible and is more cost-effective than using a single renewable energy resource. Muhammad and Kamran [10] performed an experimental study on a building-integrated photovoltaic system with a novel bi-reflector PV system (BRPVS). The proposed BRPVS showed better performance with a stable output voltage despite larger variations in input voltage.

Sun et al. [11] studied the application of heuristic optimization for grid-interactive nZEB through a glowworm swarm algorithm. The results from the study suggested that the baseline design could be outperformed by the optimized design in terms of operating costs and grid energy consumption. Li et al. [12] developed an optimal design of nZEB based on multi-stage design optimization. The results from the study showed that the proposed coordinated design method could offer optimal designs efficiently and robustly. The authors of [13] evaluated the performance of nZEB in a hot and humid climate. The proposed model showed a 37% to 50% increase in performance. Lagrange et al. [14] developed a sustainable microgrid for a hospital facility to increase power resilience in critical situations. As a result, a microgrid comprising photovoltaic panels and a diesel generator with an energy storage system was developed which could save the hospital a sum of $440,191 over 20 years with a resilience time of 34 h.

Moser and Muschick [15] performed sensitivity and performance analyses on mixed-integer linear programming based (MILP)-based modular energy management system for urban societies. The authors concluded that the annual cost could be reduced by 3 to 6% with proposed energy management system (EMS) with supplementary elements such as thermal energy storage and a battery system. Elkadeem et al. [16] proposed a grid-isolated hybrid renewable energy system for the electrification of agriculture and irrigation area. The authors carried out a case study in Sudan to demonstrate the cost of energy and the net present cost of the system in the selected location. Many other researchers [17,18,19,20,21,22,23] have succeeded in developing sustainable nZEB models but none of the models used hydroelectric energy as a renewable energy resource in a grid-isolated system.

Hydroelectric power technologies are on an exponential rise as they are playing a crucial role in minimizing the carbon footprint of the continent [24]. As far as hydropower technologies are concerned, conventional hydropower technologies have been rendered useless for application in gravity-fed vertical water channels or pipelines because in pipelines 95% of the pressure of the fluid is depleted and pipelines usually require bypass loops to avoid the depletion of pressure. Therefore, for gravity-fed vertical pipeline systems, specialized turbines with proper design considerations may be used.

A water rotor or hydro-turbine is a mechanical device that translates the kinetic energy of moving water to rotational mechanical energy to generate electrical energy by coupling a generator [25]. According to different rotational directions concerning the direction of flow, hydro-turbines can be categorized as cross-flow turbines and axial-flow turbines [26]. The main difference between cross-flow turbines and axial flow turbines is that cross-flow turbines do not depend on the direction of fluid flow and generally, the direction of flow is perpendicular to the axis of rotation of the turbine, design complexity level is also relatively low [27].

The Gorlov turbine, the Savonius turbine, and the Darrieus turbine are the most widespread crossflow turbines in terms of their applications. The Savonius turbine is one of the oldest types of turbines with a simple design and has applications mostly in wind energy [28]. Engineer Sigurd Johannes Savonius designed and analyzed this turbine in 1922 for the application in wind power. The fundamental design of the Savonius turbine is comprised of two semi-circular blades to form an S-shaped cross-section slightly overlapped on one another. The Savonius turbine’s rotation is independent of fluid direction, and it has reasonable starting torque even at lower speeds. However, Savonius turbines are impractical for large-scale applications, as they cannot be connected with a generator because of low efficiency and tip to speed ratio.

Georges Jean Marie Darrieus designed the Darrieus turbine in 1931 [29]. The direction of fluid is always normal to the axis of rotation of Darrieus. It has dense applications in wind energy, but it also comes with some disadvantages. It is very rarely self-starting and has very low starting torque. In 1995, Alexander Gorlov patented a design of a helical crossflow turbine and he named it the Gorlov turbine [30]. It was claimed to have improvements over the Darrieus turbine. Its basic geometry was similar to the Darrieus turbine, but its profile of hydrofoil was twisted along a helix. This twist provided the Gorlov helical turbine showed better characteristics as compared to the conventional Darrieus turbine. Recently, Lucid modified the Gorlov helical turbine to develop a crossflow spherical turbine, which was optimized for gravity-fed vertical water channels. Further, numerous numerical and experimental studies have been carried out on the aforementioned turbines to enhance their performance and design parameters.

A detailed experimental study has been carried out by Golecha et al. [31] on an adapted Savonius turbine that spans the selection of the optimum location of the deflector plate. The authors calculated the power coefficient of the turbine for different positions of the deflector plate. They conclusively explained the optimum position of deflector plate for different types of Savonious hydro-turbines and improved the power coefficient by up to 50%. Bianchini et al. [32] performed two-dimensional computational fluid dynamics (CFD) simulations on the Darrieus turbine and noted that a two-dimensional simulation of the turbine provides much better conditions for the assessment of performance characteristics of the turbine. The authors of [33] performed a numerical analysis on a vertical-axis tidal turbine with deforming blades. The authors were able to improve the relative hydrodynamic efficiency of the turbine by controlling the size and time of formation of the leading-edge vortex by the introduction of a well-imposed deflection. Yang et al. [34] studied a hunter turbine and investigated the hydrodynamic characteristics of fluid flow around it for tidal currents using two-dimensional CFD simulations. As a result, the authors concluded that the hunter turbine provides very reasonable performance over a restricted range of flow coefficient. Bachant et al. [35] carried out a study to compare the performance of the cylindrical Gorlov helical hydro-turbine and the Lucid spherical hydro-turbine. Results showed that for a low blockage channel environment the performance characteristics of a cylindrical Gorlov turbine are better lucid spherical turbine.

Derakhshan et al. [36] investigated the hunter tidal hydrokinetic turbine by performing numerical analysis on the turbine. The authors designed an experimental setup to study the velocity distribution of the fluid flow around the turbine. This study proved the direct relationship between the power coefficient of the hunter turbine and the cross-sectional area of the duct. Besides, the authors noted that the efficiency is maximum when the distance between the two consecutive turbines is thirteen times the diameter of each turbine in a four-turbine farm. The authors of [27] put forward a numerical study of a vertical-axis hydro-turbine. In this study, the three-dimensional effects are modelled on a vertical-axis cross-flow turbine. The results of this study proved the significance of three-dimensional effects for a turbine with a height less than two times its radius. However, for a turbine with a height more than seven times the radius, the three-dimensional effects can be neglected. Velasco et al. [37] used ANSYS Fluent 15.0 to carry out a numerical study to investigate the flow control based on synthetic jets for the Darrieus turbine. The study proved that, by using active flow control with synthetic jets, the performance of the Darrieus turbine can be significantly boosted for operations at a low tip to speed ratio. Elbatran et al. [38] performed a numerical study on a Savonius hydro-turbine with a ducted nozzle. This study proved that the performance of a Savonius turbine can be increased by 78% just by using the ducted nozzle.

Shimokawa et al. [39] performed an experimental study to investigate the performance of a Darrieus hydro-turbine with an inlet nozzle. The authors noted a significant increase in the performance of the Darrieus hydro-turbine by connecting the inlet nozzle at the upstream of the runner. Kaprawi et al. [40] studied a combined Darrieus–Savonius hydro-turbine. The authors proved that the combined Darrieus-Savonius hydro-turbine provides significantly better performance than a solo Savonius rotor. Sarma et al. [41] presented a numerical and experimental study on a Savonius hydrokinetic turbine. The authors analyzed the performance of the Savonius hydrokinetic turbine under a low-velocity condition in ANSYS 14.0. The authors of [42] analyzed the performance of two counter-rotated vertical-axis hydrokinetic turbines (VAHKT). They proved by the application of the ALE-VMS technique, that the performance characteristics are better when there is a distance equal to 4 times the diameter of turbines between both turbines.

Payambarpour et al. [43] performed a numerical and experimental study on an in-pipe Savonius hydro-turbine with a deflector. They concluded that the performance of a Savonius turbine could be improved by using a deflector up to some extent. Further increasing the deflector parameters affects the performance of the turbine adversely. Nishi et al. [44] studied the effect of flow rate on the performance of an undershot cross-flow hydro-turbine. Their study showed that the second-stage crossflow hydro-turbine produced a dominant torque. Shahsavarifard et al. [45] studied the performance characteristics of shrouded horizontal-axis hydrokinetic turbines (HAKT) in yawed conditions. The results from their study showed that the maximum power of a shrouded turbine is inversely proportional to the cosine of yaw angle. Ansarifard et al. [46] used ANSYS CFX 18.0 to optimize the design of a purely radial turbine for an oscillating water channel. The designed turbine achieved 81% efficiency when operated in steady mode. Langroudi et al. [46] modelled and performed a numerical and experimental study on a cross-flow turbine for the horizontal channel using ANSYS Fluent. The authors achieved significant performance improvements in the turbine design.

Researchers have implemented various design implications to improve the performance of crossflow turbines for different types of channels and flow. Most of the authors concluded that a lucid spherical turbine is the most efficient choice for a gravity-fed vertical pipeline channel.

This research paper proposes an HRES-based nZEB model for an academic building, which has a continuous flow of water and is located in an area with plenty of sunlight throughout the year. An off-grid renewable microgrid has been designed comprising photovoltaic and hydroelectric resources. The economic feasibility analysis and simulations are performed in a commercial software called HOMER Pro. The results have suggested that a building can be made energy autonomous with the help of indigenously designed hydro-turbines installed in vertical water delivery channels and photovoltaic systems.

This paper has been organized in the following manner: Section 2 explains the proposed system from the perspective of nearly Zero Energy Buildings. It also describes the methodology carried out to design and model the HRES microgrid for the system. Section 3 explains the important parameters on which the turbine is designed in SolidWorks 2017. Section 4 follows up with the analytical analysis of the turbine in ANSYS Fluent 18.1 based on the K-epsilon turbulence model. It explains the various equations needed to be satisfied for the analysis. Section 5 discusses the results of the simulations and analysis carried out for this study. Finally, Section 7 concludes the findings of this research.

## 2. Proposed System

**Figure 2:**
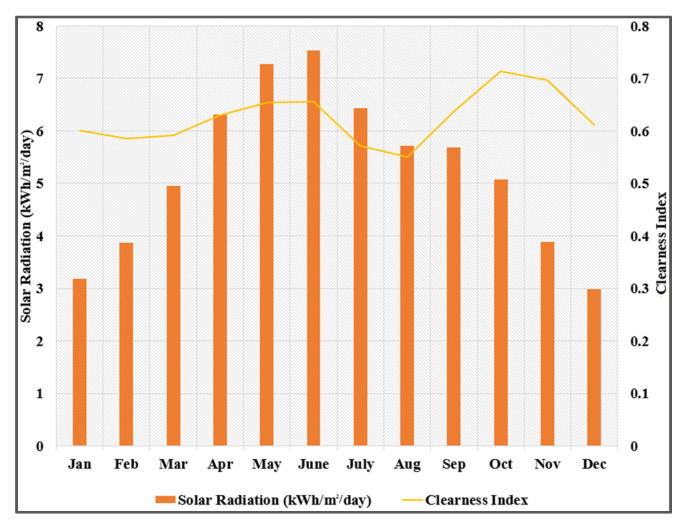
The average annual solar radiation in Islamabad.

The off-grid renewable microgrid for the nZEB model in this study is designed and optimized in HOMER Pro software. The building selected for initial testing and prototyping is an academic building with some faculty offices located in Air University Islamabad. It is a three-story building with a basement and area of approximately 4800 square feet. The electrical load is mostly general utility as the building is constructed on the principles of passive heating and cooling so there are minimal electrical cooling and heating appliances. Islamabad has a high average annual rainfall of 1142.1 mm and 3000 to 3300 annual sunshine hours. Therefore, hydroelectric and photovoltaic hybridization is fairly efficient in the city. Figure 2 shows the graphical representation of the available annual average solar radiations and clearness index in Islamabad throughout the year. This data were downloaded from the National Aeronautics and Space Administration (NASA) Surface Meteorology and Solar Energy database by HOMER.

The main building under consideration is suggested to have a water supply tank for the whole university on its roof, with vertical water delivery pipelines running from the roof to the ground. The turbines are installed in the pipe and adjacent turbines are 1 foot apart. In this way, each pipe can house up to 23 turbines and the effective head for each turbine is 1 foot. The design of turbine is explained in Section 3. To rectify the noise generated by excessive moving parts, noise dampeners are used around the pipes. 

The water reservoir located on the rooftop has a height of 4.2 m and the orifice for the pipe at the bottom has an internal diameter of 6.6 inches. If the coefficient of discharge is taken as 0.975 and height of the water above the orifice is taken as 4 m, the water discharge velocity is calculated as 10.45 m/s according to the relation in Equation (1) [47,48].

(1) $$v=C_{d}\times \sqrt{2gH}$$

where v is the velocity of fluid, $C_{d}$ is the coefficient of discharge, g is the gravitational acceleration and H is the height of fluid above the discharge orifice. As the diameter of the pipe is smaller than the orifice, 6 inches to be precise, the velocity of the fluid is increased as per the equation of continuity (Equation (2)). The velocity in the pipe is calculated to be 12.66 m/s [47,48].

(2) $$A_{1}v_{1}=A_{2}v_{2}$$

$A_{1}$ and $v_{1}$ represent the cross-sectional area of fluid channel and velocity of fluid at A position while $A_{2}$ and $v_{2}$ represent the cross-sectional area of fluid channel and velocity of fluid at B position. Finally, the water flow rate is calculated as 230.9 dm^3^/sec for the simulation. 

**Figure 3:**
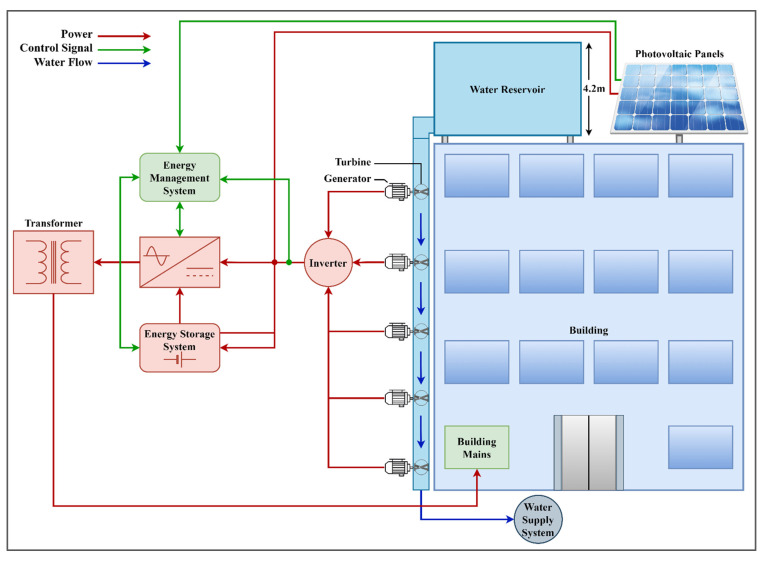
Complete schematic of proposed nZEB.

The roof of the building is also flat and shadow-free which allows for the installation of high-efficiency monocrystalline photovoltaic panels on the roof. The complete schematic design of the building is shown in Figure 3. The electrical power from the hydro-turbines and photovoltaic panels is converted to AC through an inverter. The energy management system fetches the data from the resources and energy storage system to optimize the system for cost and efficiency.

### 2.1. Load Profile

**Figure 4:**
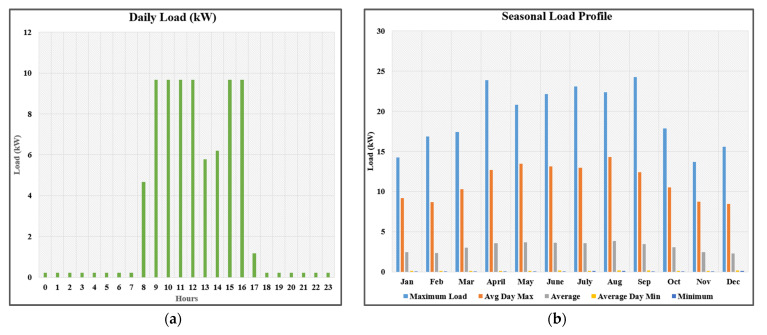

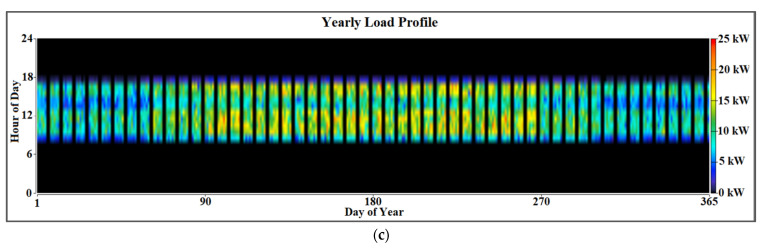
(**a**) Daily load profile; (**b**) annual load profile; (**c**) hourly load profile throughout the year.

A custom load profile for a university facility is built by observing the actual load of the building for 6 months and extrapolating the results to one year. The load is scaled down to accommodate all the sensitivity cases in HOMER Pro. The load is taken as 18.68 kWh/day with a peak of 6.07 kW and the load factor is 0.13. The load starts to rise from 08:00 am when the office time starts, and it declines down at 01:00 pm due to the lunch break. There is negligible load from 06:00 pm to 07:00 am. As the building houses the faculty offices only so no exam breaks, or annual breaks are observed Figure 4a–c show the daily load profile, annual load profile, and hourly load profile throughout the year, respectively.

### 2.2. Microgrid Design

**Figure 5:**
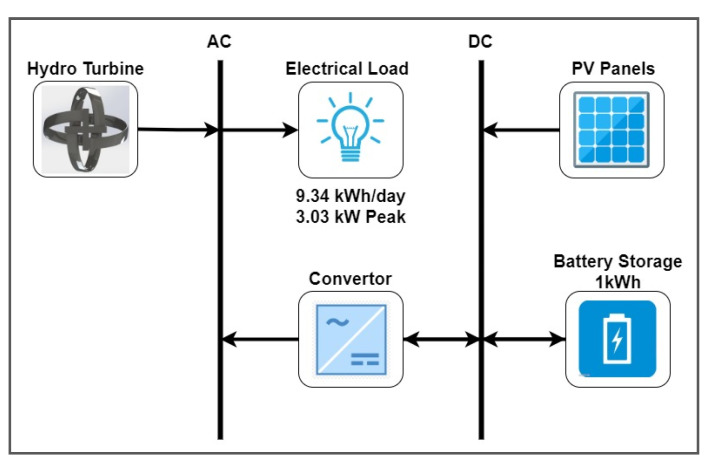
Microgrid configuration designed in HOMER.

An AC/DC microgrid is designed in HOMER with parameters of the designed hydro-turbine and solar photovoltaic as renewable energy resources and load with a load profile discussed earlier. 1 kWh, 83.4 Ah lead-acid battery units are also added for energy storage and stability of the system. DC bus is energized through PV Panels and a hydro-turbine. There is a two-way connection between the lead-acid battery and the DC bus to facilitate the charging and discharging of batteries. A DC to AC converter is connected between DC and AC buses. The electric load is powered through an AC bus. It is a standalone system, and no non-renewable energy resource is utilized. Figure 5 shows a simplified schematic diagram of the microgrid developed in HOMER.

Table 1 and Table 2 list all the parameters for solar photovoltaic panels and lead-acid batteries, respectively, used in the simulation.

### 2.3. Optimization

HOMER simulates all the hybrid energy systems possible and finds the optimal hybrid energy system through applying optimization algorithms on the systems continuously and repeatedly in real time. Several constraints such as solar radiations and water flow are analyzed and simulated for the best optimum economic strategy and the best combination of resources are provided based upon the optimizations [49].

The most cost-effective energy system architecture is determined and analyzed in terms of *COE* and *NPC*. HOMER shows a rundown of all the possible architectures that can meet the load demand. The *COE* and *NPC* of all the architectures is the key requirement to carry out a techno-economic analysis. The *COE* for all the architectures is calculated by HOMER through Equation (3).

(3) $$COE=\frac{C_{TANN}}{E_{ls}+E_{Grid}}$$

where ‘$C_{TANN}$’ denotes the total annual cost of the system, $E_{ls}$ defines the total energy delivered by the hybrid energy system (HES) to the load side and $E_{Grid}$ is the energy supplied to the grid. In this case, all the arrangements are off-grid so ‘$E_{Grid}$’ will be zero. The net present cost (*NPC*) of the system includes capital cost, installation cost, and operational costs over a time period, which is 20 years in this case. The net present cost (*NPC*) is determined through Equation (4).

(4) $$C_{NPC}=\frac{C_{TANN}}{CRF_{\left(i,N\right)}}$$

where ‘$C_{TANN}$’ is the total annual cost, ‘*i*’ is the total interest rate over the time period, and ‘*N*’ represents the number of years over which interest is applicable. The interest rate is fixed at 5% and *CRF* is the acronym for ‘capital recovery factor’, which is the ratio of capital cost to the received annuity in the cash flow. It is calculated through Equation (5).

(5) $$CRF_{\left(i,N\right)}=\frac{i{\left(1+i\right)}^{N}}{{\left(1+i\right)}^{N}-1}$$

## 3. Turbine Design

As the designed microgrid needs an indigenously designed gravity fed turbine for small-scale production, a turbine is designed based on numerous parameters. Power is one of the most important of them. The turbine is mainly designed on the power of flowing fluid, from which power is to be extracted [50]. Therefore, a thorough understanding of the available power of the flow medium is required. This power is calculated by the relationship given in Equation (6). It is the measure of the kinetic energy of the fluid with respect to time.

(6) $$P_{available}=\frac{1}{2}\rho A_{c}U_{\infty }^{3}$$

where ρ is the density of the fluid, $A_{c}$ represents the area of the cross-sectional area of turbine and $U_{\infty }$ is the free-stream velocity of the fluid. Equation (4) shows that the power of the fluid of density ρ flowing through a channel is directly proportional to the cube of free-stream velocity ($U_{\infty })$ of the fluid. The hydrodynamics of a crossflow turbine especially, a lucid turbine are complex and dependent on numerous factors. The flow passing through a crossflow turbine is highly unsteady and turbulent. This unsteady flow is the result of the interaction of shed vortices created on the upper side of the turbine by its blade, which affects the forces on the lower side of the turbine negatively. Moreover, the constantly changing angle of attack on the turbine blades throughout the rotation also results in a dynamic stall, which affects the turbine. Keeping in mind all these problems and complexity in the turbine design, the application Blade Elemental Momentum (BEM) theory is the recommended strategy. BEM theory helps calculate all the local forces and induced velocities on the turbine blades.

**Figure 6:**
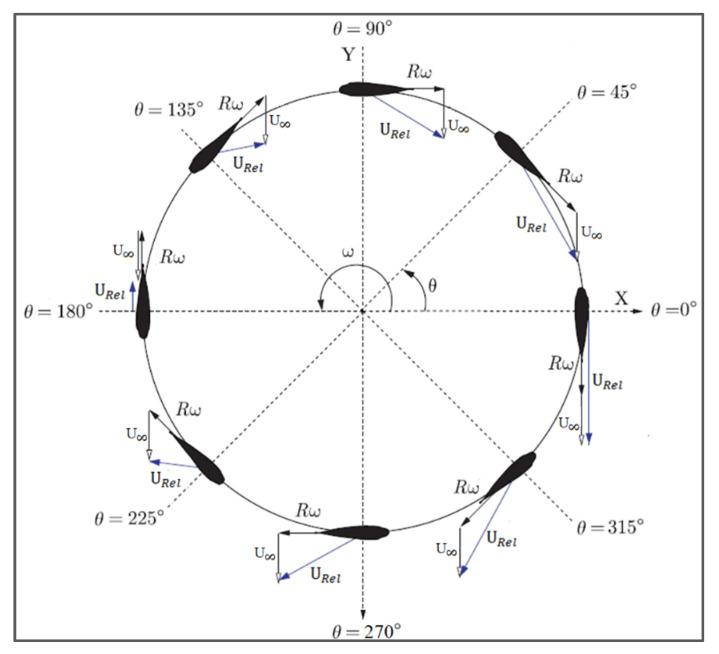
Velocity triangles for the spherical helical turbine in a gravity-fed system.

Figure 6 shows the possible velocities induced in the turbine’s blade. The turbine’s blade is divided into infinitely small cross-sections at regular intervals in its path of rotation and induced velocities are observed in all cross-sections. As a result, velocity triangles are formed as shown in Figure 6. Azimuthal coordinate *ϴ* represents the blade position. As the azimuthal coordinate is changing, the angle of attack (α) is changing, and consequently, blades of the turbine experience the resultant velocities (*U_Rel_*).

*U_Rel_* is the resultant velocity which is the vector sum of free stream velocity of flow medium ($U_{\infty }$) and that is created from the free-stream ($U_{\infty }$) and rotational velocity (Rω). The resultant velocity *U_Rel_* can be illustrated from Equation (7).

(7) $$U_{Rel}=\sqrt{{\left(U_{\infty }+\omega Rcos\theta \right)}^{2}+{\left(\omega Rsin\theta \right)}^{2}}$$

where *U_Rel_* is the relative velocity of the turbine, $U_{\infty }$ is the free stream velocity of the fluid, *R* represents the radius of the turbine, *ω* is the rotational speed of the turbine and *θ* is the azimuthal angle. By establishing a relationship between tip-to-speed ratio and resultant velocity (*U_Rel_*), the angle of attack can be determined. The angle of attack (α) is calculated through Equation (8).

(8) $$\alpha =\frac{sin\theta }{cos\theta +\lambda }$$

where *α* represents the angle of attack of the blade and *λ* is the tip-to-speed ratio.

### 3.1. Blade Profile

Turbine blades hold significant importance in a turbine design. They are the mean of extraction of energy from the fluid. The fluid flow applies the forces on the blades at the center of pressure of the hydrofoil profile. The blade experiences both normal and tangential forces exerted by the fluid which results in a greater lift force to drag force ratio on the blade. This phenomenon is the main reason for the rotation of the turbine. This geometry of the forces is shown in Figure 6.

**Figure 7:**
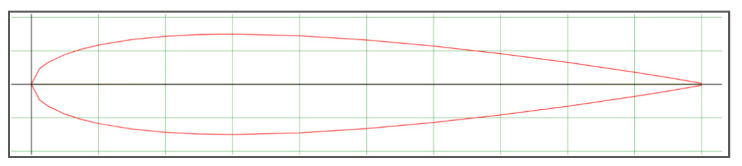
NACA 0015 blade profile for hydro-turbine [51].

NACA 0015 blade profile commissioned by National Advisory Committee for Aeronautics (NACA) has been used in the design of this turbine. The blade profile of NACA 0015 is shown in Figure 7.

**Figure 8:**
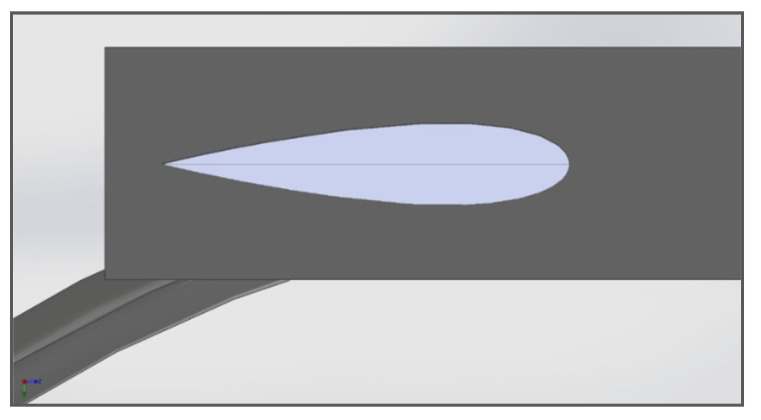
Blade profile of turbine based on the NACA 0015 design.

Figure 8 shows the cross-section of the actual hydrofoil profile used in the design of the turbine. The hydrofoil design is symmetrical, i.e., the total area above and below the camber line is equal.

### 3.2. Tip-to-Speed Ratio

Tip to speed ratio (*λ*) is one of the major parameters, on which a turbine is designed. It is the ratio of the turbine’s rotational speed relative to the laminar speed of the fluid. It is defined by the rate of change of angle of attack on the turbine blade and it is the parameter, which defines the power coefficient of a turbine. It is expressed by the Equation (9).

(9) $$\lambda =\frac{\omega R}{U_{\infty }}$$

### 3.3. Torque and Power Coefficient

The ratio of torque generated by the turbine under the action of flow to the total thrust generated by the fluid along the radius of the turbine if all the kinetic force of the fluid is considered concentrated from the full radius of the turbine. The torque may be taken as the cross product of the force, which acts at the center of pressure of the hydrofoil and radius of the turbine. Equation (10) represents the torque coefficient.

(10) $$C_{Q}=\frac{2T}{\rho U_{\infty }^{2}RA_{c}}$$

where *C_Q_* represents the coefficient of torque, *T* is the net torque on the turbine, $A_{c}$ is the turbine cross-sectional swept area and ρ is the density of the fluid.

Power coefficient is also a very important hydrodynamic for a turbine. Power coefficient (*C_p_*) is the ratio of total power extracted from the turbine to the theoretical power of the fluid resource. This dimensionless quantity can also be represented in form of torque coefficient and tip-to-speed ratio. The power coefficient can be expressed through Equation (11).

(11) $$C_{p}=C_{Q}\lambda$$

### 3.4. Final Design

A conceptual design of the turbine is constructed in SolidWorks 2017 with all the parameters and hydrodynamics of the turbine discussed above. A turbine with four blades with a chord length of approximately 15 mm, a turbine height of 107 mm, and turbine diameter of 115.6 mm are designed. These values were selected to accommodate a small-scale turbine in a vertical cylindrical channel of diameter 152.4 mm for real-time experimental testing. The parameters are later justified by numerical analysis in ANSYS Fluent 18.1. Table 3 represents all the design parameters of the spherical helical turbine, which are implemented to design the turbine in SolidWorks 2017. The turbine perfectly fits in a cylindrical channel of the diameter of 6 inches or 152.4 mm without making a hindrance in the flow of fluid around the corners of the turbine.

**Figure 9:**
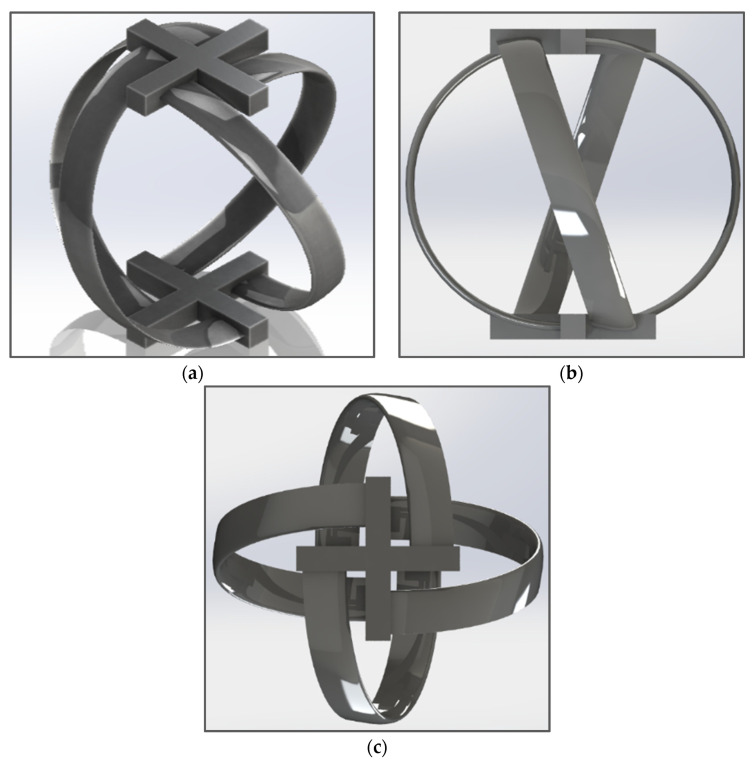
(**a**) Oblique view of the turbine geometry; (**b**) front view of the turbine geometry; (**c**) top view of the turbine geometry.

Figure 9 shows different views of the spherical helical turbine. The turbine features a NACA 0015 blade profile. The material of the turbine modelled in SolidWorks 2017 is polished aluminum to retain minimum skin friction, which allows maximum turbulent kinetic energy to be transferred to the turbine.

## 4. CFD Analysis

### 4.1. Turbine Container

**Figure 10:**
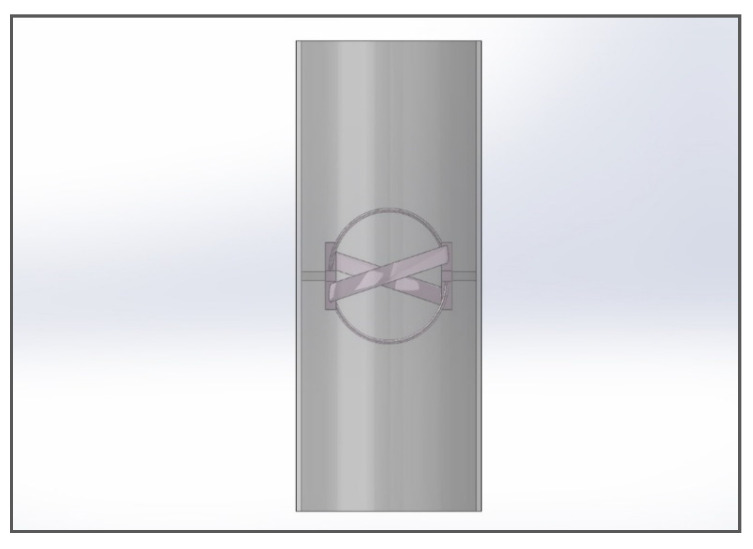
The turbine assembly with the pipe.

For the sake of CFD analysis, the turbine is considered to be assembled in an aluminum pipe with an internal diameter of 6 inches or 152.4. The pipe walls have a smoothness factor δ = 1. The distance between the walls of the pipe and the turbine top and bottom is kept to be 22.7 mm to ensure smooth rotation of the turbine without increasing the friction between fluid and turbine walls. The assembly shown in Figure 10 depicts the actual arrangement of the turbine. This assembly is then subjected to meshing and CFD analysis.

### 4.2. Meshing

The computational domain of the turbine is imported into a commercially available software Geometry and Mesh Building Intelligent Tool (GAMBIT) to generate the mesh. A non-conformal unstructured mesh is used to generate mesh for the entire computational domain characterized by the tetrahedral elements. A prismatic mesh layer is generated in the turbine blade to capture rapid variations in the pressure, the velocity, and the turbulent kinetic energy of the flow around the turbine.

**Figure 11:**
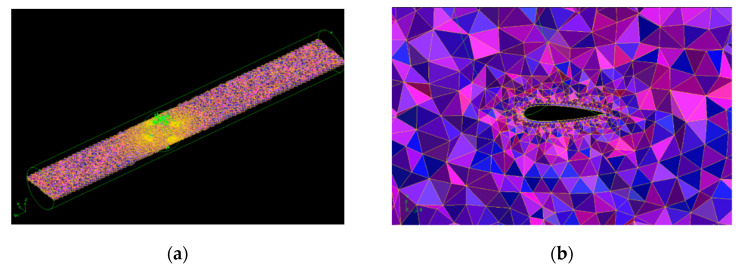

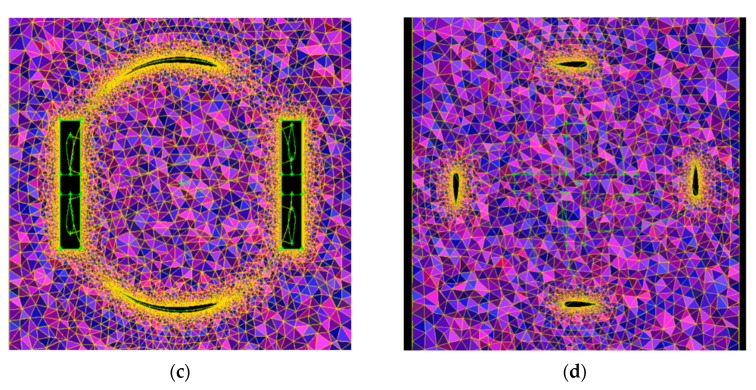
(**a**) Mesh of the turbine and surrounding space around the turbine; (**b**) mesh of a single blade of the turbine; (**c**) static mesh of fluid around turbine in lateral view; (**d**) static mesh of fluid around turbine in top view.

Zhou et al. [52] suggested that in order to use the realizable *k−ε* turbulence model, a y+ value less than one is recommended to calculate the distance of the first mesh node from the turbine blades surfaces. Therefore, a y+ value less than one is chosen in the present study. Finally, a skew-able mesh for both rotating and fixed domains is developed in GAMBIT with an aspect ratio of 1:0.00045. Figure 11a shows the mesh inside the channel, Figure 11b shows the mesh around a single blade, and Figure 11c,d show the static mesh of fluid around the turbine in different perspectives.

**Figure 12:**
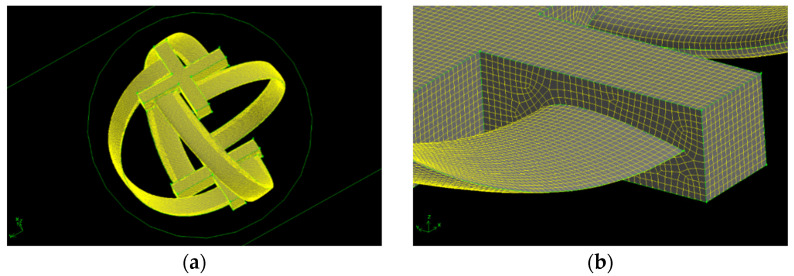
(**a**) Dynamic mesh of turbine walls; (**b**) zoomed-in representation of the turbine’s dynamic mesh.

The turbine walls are processed as a dynamic mesh because of rotatory mechanics. The mesh of the whole turbine is shown in Figure 12a and a zoomed-in perspective is shown in Figure 12b. The size of the dynamic mesh is kept constant to avoid complexity in the mesh. The dynamic zone is represented by a green circle around the turbine. The turbine is rotating inside this circle.

### 4.3. Numerical Analysis

The common practice for analyzing a turbine in a dynamic rotating environment is solving the turbulent model in ANSYS Fluent, as apparent in the literature review [37,41,46]. Therefore, the turbine is subjected to numerical analysis in ANSYS Fluent 18.1 to solve the unsteady incompressible Reynolds-averaged Navier–Stokes equations. The Navier–Stokes equations can be expressed by the Equations (12)–(14) [53,54].

(12) $$\frac{\partial \rho }{\partial t}+\frac{\partial \left(\rho u_{i}\right)}{\partial x_{i}}=0$$

(13) $$\frac{\partial \left(\rho u_{i}\right)}{\partial t}+\frac{\partial \left(\rho u_{i}u_{j}\right)}{\partial x_{j}}=-\frac{\partial p}{\partial x_{i}}+\frac{\partial }{\partial x_{j}}\left[\mu \left(\frac{\partial u_{j}}{\partial x_{j}}+\frac{\partial \left(u_{j}\right)}{\partial x_{i}}-\frac{2}{3}\delta_{ij}\frac{\partial u_{i}}{\partial x_{i}}\right)\right]+\frac{\partial \left(-\rho u_{i}^{\prime }u_{j}^{\prime }\right)}{\partial x_{j}}+F_{i}$$

where

(14) $$-\rho u_{i}^{\prime }u_{j}^{\prime }=\mu_{t}\left(\frac{\partial u_{i}}{\partial x_{j}}+\frac{\partial u_{j}}{\partial x_{i}}\right)-\frac{2}{3}\rho k\delta_{ij}$$

The quantity ‘$-\rho u_{i}^{\prime }u_{j}^{\prime }$’ the turbulent stress, ‘*p*’ is the pressure, ‘*u*’ is the velocity of the fluid, ‘*Fi*’ is a vector representing the external forces and ‘*ρ*’ is the density of the water. The *k*−*ε* turbulent model is chosen to solve the Navier–Stokes equations in ANSYS 18.1. The *k*−*ε* model can be explained by Equations (15) and (16).

(15) $$\frac{\partial }{\partial t}\left(\rho k\right)+\frac{\partial }{\partial x_{i}}\left(\rho ku_{i}\right)=\frac{\partial }{\partial x_{j}}\left(\alpha \mu_{\mathrm{eff}}\frac{\partial k}{\partial x_{j}}\right)+G_{k}+G_{b}-\rho \varepsilon -Y_{M}+S_{k}$$

(16) $$\frac{\partial }{\partial t}\left(\rho \varepsilon \right)+\frac{\partial }{\partial x_{i}}\left(\rho \varepsilon u_{i}\right)=\frac{\partial }{\partial x_{j}}\left(\alpha \mu_{\mathrm{eff}}\frac{\partial \varepsilon }{\partial x_{j}}\right)+C_{1\varepsilon }\frac{\varepsilon }{k}\left(G_{k}+C_{3\varepsilon }G_{b}\right)-C_{2\varepsilon }\rho \frac{\varepsilon^{2}}{k}-R_{\varepsilon }+S_{\varepsilon }$$

where ‘*G_b_*’ represents the turbulent kinetic energy generated due to the buoyancy, ‘*G_k_’* represents the turbulent kinetic energy generated due to the mean velocity gradients of the fluid and ‘*Y_M_*’ presents the contribution of the fluctuating dilatation in compressible turbulence to the overall dissipation rate. ‘*α_k_*’ and ‘*α_ε_*’ represent the inverse of the effective Prandtl numbers for *k* and *ε,* respectively. ‘*S_k_*’ and ‘*S_ε_*’ are the user-defined source terms. The modelled transport equations for *k* and *ε* in the realizable *k*−*ε* model are presented by Equations (17) and (18).

(17) $$\frac{\partial }{\partial t}\left(\rho k\right)+\frac{\partial }{\partial x_{ij}}\left(\rho ku_{j}\right)=\frac{\partial }{\partial x_{j}}\left[\left(\mu +\frac{\mu_{t}}{\sigma_{k}}\right)\frac{\partial k}{\partial x_{j}}\right]+G_{k}+G_{b}-\rho \varepsilon -Y_{M}+S_{k}$$

(18) $$\frac{\partial }{\partial t}\left(\rho \varepsilon \right)+\frac{\partial }{\partial x_{j}}\left(\rho \varepsilon u_{j}\right)=\frac{\partial }{\partial x_{j}}\left[\left(\mu +\frac{\mu_{t}}{\sigma_{\varepsilon }}\right)\frac{\partial k}{\partial x_{j}}\right]+C_{1\varepsilon }\frac{\varepsilon }{k}\left(G_{k}+C_{3\varepsilon }G_{b}\right)-C_{2\varepsilon }\rho \frac{\varepsilon^{2}}{k}-R_{\varepsilon }+S_{\varepsilon }$$

where

(19) $$C_{1}=max\left[0.43,\;\frac{\eta }{\eta +5}\right]$$

(20) $$\eta =S\frac{k}{\varepsilon }$$

(21) $$S=\sqrt{2S_{ij}S_{ij}}$$

After choosing the turbulence model for the numerical analysis, the analysis is carried out in ANSYS 18.1 with boundary conditions. Convergence criteria is considered for continuity, momentum, and turbulence characteristics for each time step. The time step is defined as 1°/time step, which means in each time step the rotor turned 1°. The considered time step size of 0.00333 s is employed.

**Figure 13:**
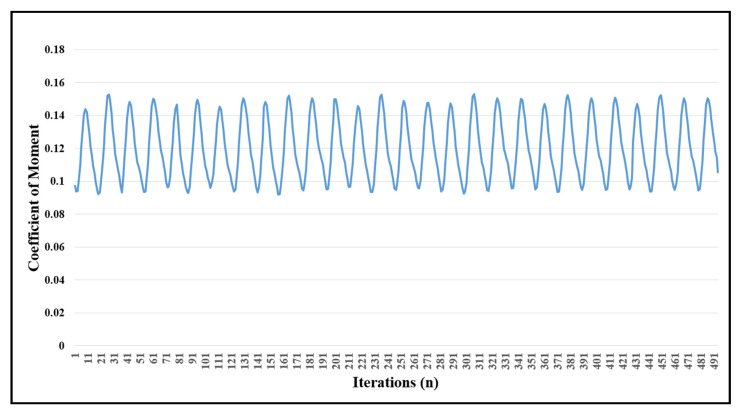
Graphical representation of the coefficient of momentum.

Even after 491 iterations, the solution remains positive, and the results gave a nearly stable co-efficient of the moment (*C_m_*) of turbine indicating minimum fluctuations in turbine’s torque and angular speed. The plot in Figure 13 represents the moment of turbine plotted in 491 iterations. Each complete cycle represents a 360° rotation of the turbine in simulated conditions. The mechanical power of the designed turbine was finally calculated to be 168 W. The moment divided by the dynamic pressure, the area, and the chord of the airfoil gives the coefficient of the moment of the turbine as represented by Equation (22).

(22) $$C_{m}=\frac{M}{qSc}$$

where $C_{m}$ is the coefficient of the moment, *M* is the moment of the turbine, *q* represents dynamic pressure, and *S* is for wing area and *c* denotes the length of the chord. The co-efficient of power can be calculated by Equation (23).

(23) $$C_{p}=C_{m}\times \lambda$$

### 4.4. Hardware Prototype

An experimental setup is designed to verify the numerical results from ANSYS Fluent 18.1 in real time. The spherical helical turbine is 3D printed on Prusa i3 3D printer using Poly-Vinyl Alcohol (PVA) as supporting material and Poly-Lactic Acid (PLA) as principal material due to the delicate nature of the geometry. The whole process took approximately 12 h. The settings on which the turbine is constructed through 3D printing technology are given in Table 4.

**Figure 14:**
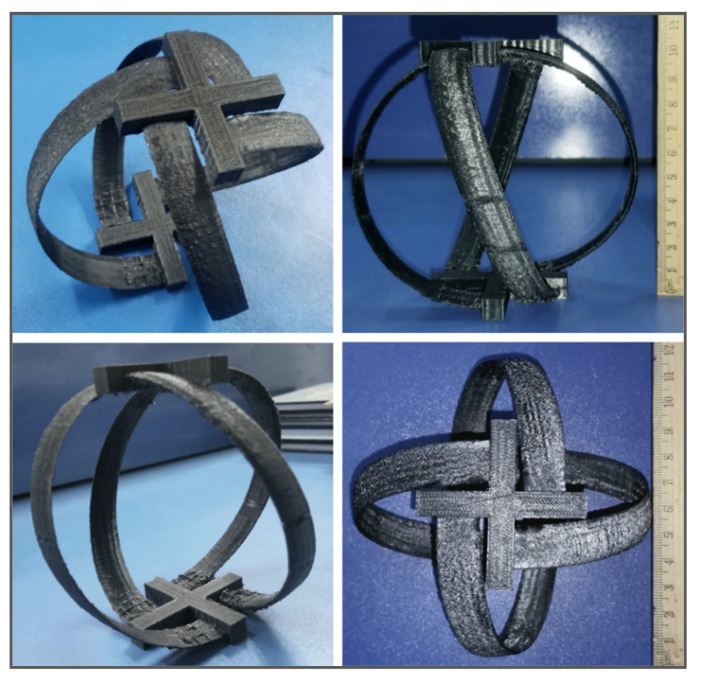
3D model of the turbine obtained through 3D printing.

**Figure 15:**
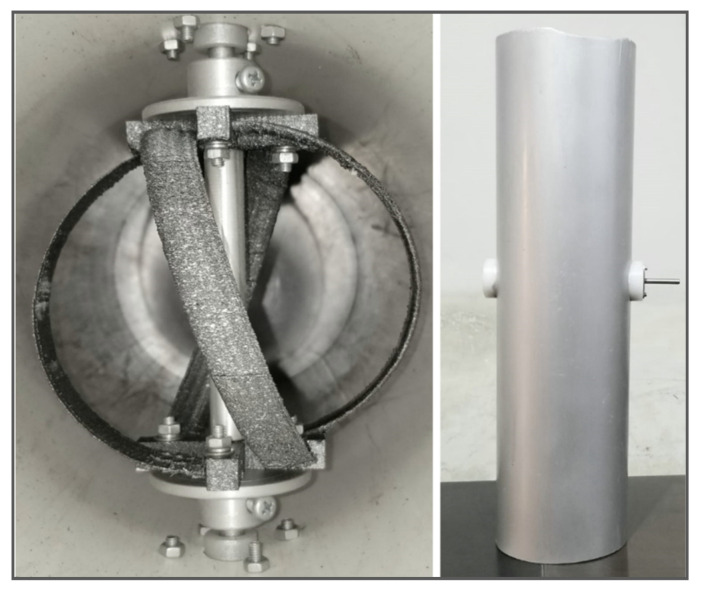
Prototype assembly and testing setup.

The final print was a 1:1 copy of the design and is shown in Figure 14. The 3D-printed model is fitted into a cylindrical channel of diameter 152.4 mm. A free shaft is connected to take measurements. A water flow of 0.231 m^3^/s is simulated, and the rotation of the turbine is studied underflow. A velocity of 231 rpm was measured through a tachometer, which is quite in conjunction with numerical results. The 3D-printed turbine is shown in Figure 14 with different perspectives and the complete setup for experimental verification is shown in Figure 15. The central aluminum rod is detachable while testing.

## 5. Results and Discussions

### 5.1. CFD Results

**Figure 16:**
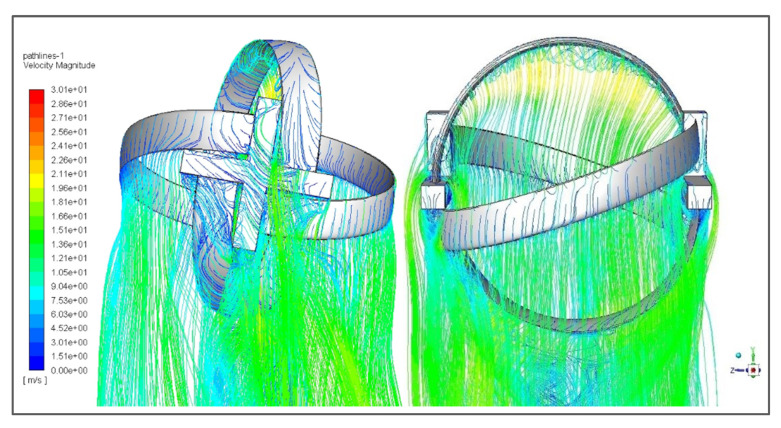
Velocity pathlines of the turbine showing the fluid particle trajectory upon impact with turbine.

CFD analysis in ANSYS Fluent 18.1 has provided various plots, contours, and pathlines to verify the feasibility and optimized operation of the turbine. Velocity pathlines of the turbine show that how the fluid particles have scattered after striking the blade surface and how the velocity of fluid particles have changed during the interaction. Velocity pathlines also show the direction of velocity by showing the scattered pattern of individual particles with respect to flow direction before impact. Figure 16 shows the velocity pathlines of the fluid after impact with the turbine. The decrease in velocity of the fluid right after impact is indicated by the pathlines.

**Figure 17:**
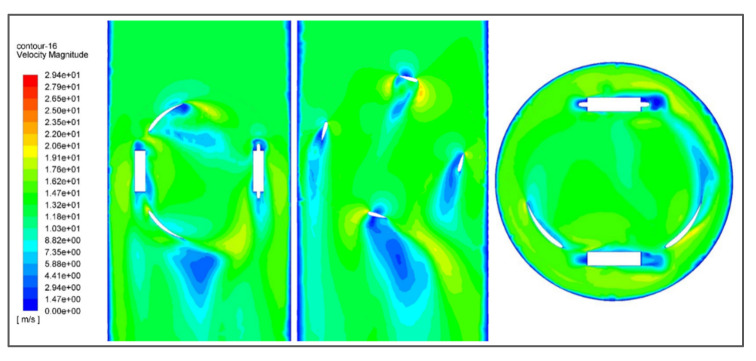
Velocity contour of the turbine showing the variation in fluid velocity along the mesh.

Velocity contours show velocity measurements translated into average cross-sectional flow velocities by contouring the point velocities. In short, they show the change in velocity magnitude with respect to flow behavior. Figure 17 shows the velocity contours of the fluid around the turbine and how its velocity changes after the impact.

**Figure 18:**
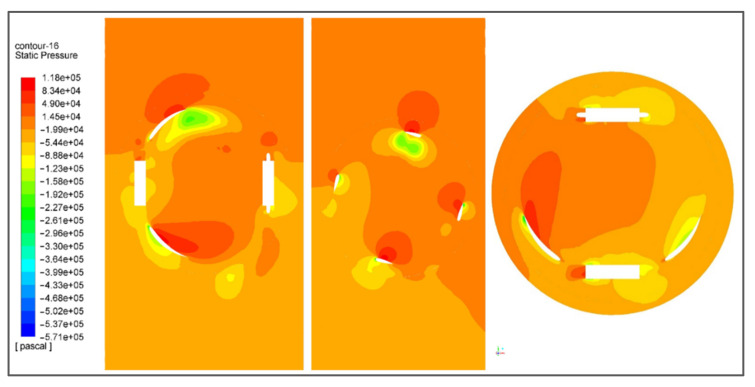
Pressure contour of the turbine showing the variation in pressure along with the mesh.

Pressure contour shows the relative change in pressure with the continuous notion. It shows the pressure gradient with respect to the flow direction before and after the impact. Pressure contours are obtained by pressure measurements translated into average cross-sectional. Figure 18 shows the pressure contour of the turbine in all views possible. It implicitly explains the change in static pressure of the fluid. It decreases significantly right after the impact with the blade, indicating the transfer of energy from fluid to the turbine.

**Figure 19:**
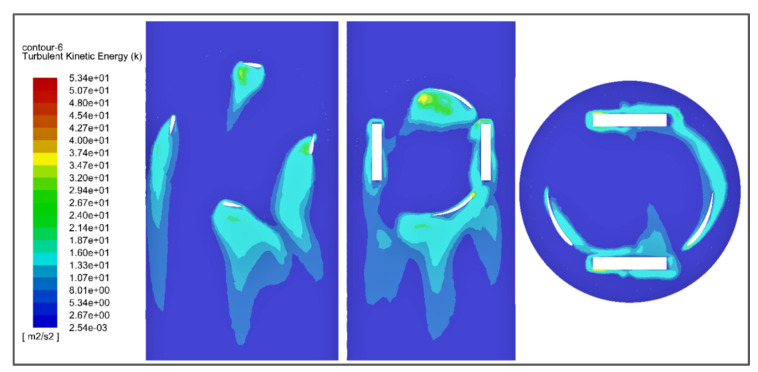
Turbulent kinetic energy contour showing the variation in turbulent kinetic energy along the mesh.

Turbulent kinetic energy (TKE) is the mean kinetic energy per unit mass associated with eddies in the turbulent flow of the fluid. Physically, the turbulence kinetic energy is characterized by measured root-mean-square (RMS) velocity fluctuations. The turbulent kinetic energy of the fluid and kinetic energy due to buoyancy are together responsible for turbine operation but in a vertical pipe turbulent kinetic energy is much greater than kinetic energy due to buoyancy. The turbulent kinetic energy contour shown in Figure 19 represents the change in TKE of the fluid at different positions of the fluid flow.

**Figure 20:**
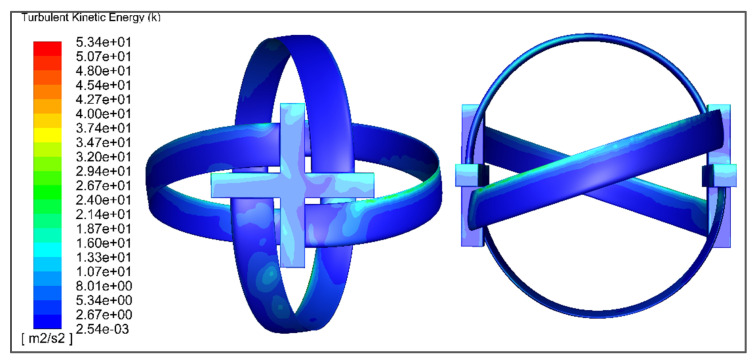
The turbulent kinetic energy of the turbine wall showing the variation in turbulent kinetic energy along the turbine wall.

The total turbulent kinetic energy on the turbine surface is characterized by the contour shown in Figure 20. It can be characterized that, at the point of impact, TKE is significantly lower.

**Figure 21:**
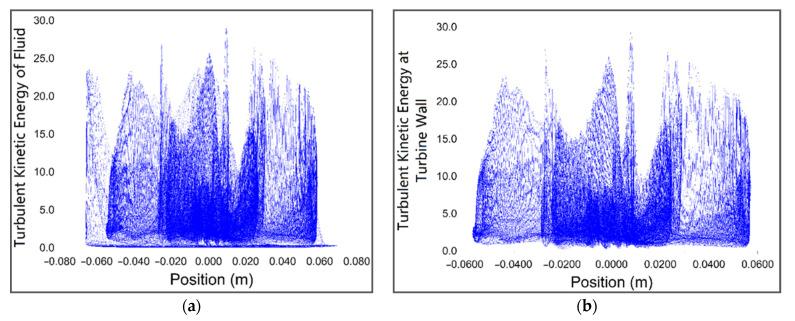

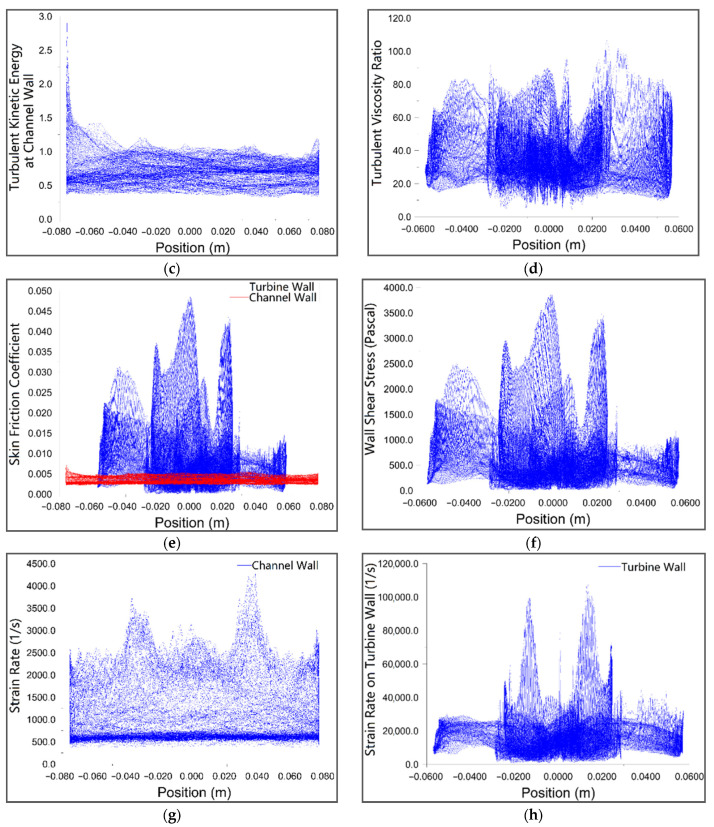
(**a**) The total turbulent kinetic energy of the fluid; (**b**) turbulent kinetic energy at turbine wall; (**c**) turbulent kinetic energy at channel wall; (**d**) turbulent viscosity ratio; (**e**) the skin friction coefficient of turbine wall and channel wall; (**f**) shear stress on the turbine wall; (**g**) strain rate at channel wall; (**h**) strain rate of the turbine.

The plot shown in Figure 21a,b represents the total turbulent kinetic energy of the fluid and turbine wall, respectively, with respect to the position. It is evident from the plot that most of the turbulent kinetic energy of the fluid is utilized in driving the turbine. The plots shown in Figure 21c,d characterizes the turbulent kinetic energy wasted by the impact of fluid with channel wall and turbulent viscosity ratio, respectively. The skin friction coefficient of the turbine wall and channel wall fluctuates against position according to the plot shown in Figure 21e.

According to the stress analysis completed in ANSYS 18.1 Fluent, it can be concluded that the turbine can easily handle the stress of 3821 Pascal with a strain rate of $1.1\times 10^{5}$ per second. The plot in Figure 21f shows the wall shear stress applied by the fluid on the turbine wall. The plot in Figure 21g,h shows the strain rate of the channel and turbine wall, respectively.

### 5.2. Techno-Economical Analysis of the nZEB Model

For an in-depth techno-economical analysis of the nZEB model, three distinct hybrid renewable energy architectures are considered and are processed through optimization algorithms in HOMER Pro. These architectures are based on the lowest cost of energy and highest stability. The architectures are photovoltaic-hydroelectric (PV-H), hydroelectric standalone (H), and PV standalone (PV).

The arrangements considered were off-grid and the extent of every design was sufficiently streamlined to deal with the load profile for the university facility. To avoid complications and grid consolidations, no additional energy is generated that can be exported to the grid and hence all the arrangements are economically viable.

All three architectures are repeatedly optimized for the lowest COE and NPC. Where the cost of energy is the per kWh cost of the energy produced and the net present cost represents the net value of the components including all the installation and capital costs. The outcome of all three systems is analyzed and compared with the base hybrid system. Table 5 represents the analysis of all three architectures with output power and rating of all components required for each system. Moreover, it shows the cost of energy and the net present cost of each architecture.

**Figure 22:**
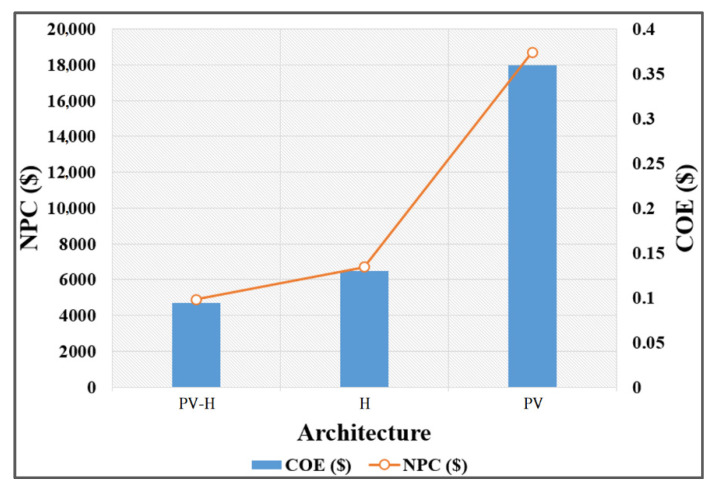
NPC and COE of selected architectures.

The COE and NPC for the PV-hydroelectric hybrid system were found to be $0.094 and $4902.807, respectively. The COE and NPC of all three architectures are compared in Figure 22. According to the plot, the COE and NPC for the standalone PV and Hydroelectric architectures are considerably higher than that of the Hybrid System, which justifies the hybrid operation of the solar and hydro-turbine for nZEB.

Table 6 shows the capital cost, replacement cost, and operating and maintenance cost of the components of the selected architecture, i.e., PV-hydroelectric hybrid system.

## 6. Limitations and Future Work

Although the microgrid is optimized for the lowest running and capital costs, the turbine delivered lower efficiency due to several factors. Firstly, it is a small-scale turbine and disturbances due to vortices affect the movement. Secondly, the effective cross-sectional area of the turbine is smaller than the actual cross-sectional area of the pipe to avoid vortices, due to which the effective fluid flow rate is reduced. These limitations should be removed by simulating the turbine in a more dynamic environment and showing more dependencies. A two-phase water model should also be used to evaluate the mechanical strength of the water. Work is also being done to find a way around the vortices problem in a small diameter pipe.

## 7. Conclusions

Hybrid renewable energy systems have the potential to revolutionize the global energy sector and can contribute substantially to the reduction in the carbon footprint from the planet. The research conducted in this study resulted in an indigenously designed hydro-turbine for a vertical pipeline system in SolidWorks 2017. The turbine is proved to be fairly efficient and provided 168 W of mechanical power with an angular speed of 100 r.p.m when analyzed with the application of the K-epsilon turbulent model in ANSYS Fluent 18.1. The detailed analysis proved that the turbine is sustainable under high stress caused by the high flow rate of the fluid. The transfer of turbulent kinetic energy from fluid to the turbine is maximum. A hardware prototype is also developed through 3D printing technology and tested in a simulated vertical cylindrical channel.

Based on the findings from the turbine, an energy management system is developed and optimized in HOMER which integrates the electrical power output from the hydro-turbine with the solar power system to achieve a self-sustained energy system for an nZEB model. A load of a four-story academic building in Air University Islamabad was considered for this energy management system. The system is comprised of hydro-turbines installed in the vertical pipelines of the building coming down from the roof and monocrystalline photovoltaic panels installed on the roof. The results obtained from the HOMER Pro proved that the HRES comprising both hydropower and solar photovoltaic power system is the best economical choice in terms of COE and NPC of the system. The selected architecture gave an average COE of $0.09418. Future research in the field of nZEB can be carried out to achieve stability and reliability in grid-isolated systems with 100% of renewable energy penetration.

## Figures and Tables

**Table 1:** Parameters of solar PV panels used in simulation in HOMER Pro.

Parameter	Specification
Panel Type	Flat Plate
Capacity	1 kW
Capital Cost	$1000
O&M Cost	$10/Year
Lifetime	25 Years
Derating Factor	80%
Ground Reflectance	20%
Nominal Operating Cell Temperature	47 °C
Temperature Effects on Power	−0.500%/°C
Efficiency at Standard Test Conditions	13%

**Table 2:** Parameters of lead-acid batteries used in simulation in HOMER Pro.

Parameter	Specification
Nominal Voltage	12 V
Nominal Capacity	1 kWh
Maximum Capacity	83.4 Ah
Capacity Ratio	0.403
Rate Constant	0.827/h
Roundtrip efficiency	80%
Maximum Charge Current	16.7 A
Maximum Discharge Current	24.3 A
Maximum Charge Rate	1 A/Ah

**Table 3:** Design parameters of the spherical helical turbine.

Parameters	Specification
Torque (T)	6.417 Nm
Angular Velocity (ω)	26.18 rad/s
Tip to Speed Ratio (λ)	0.1195
Blades (B)	4
Height (H)	107.14 mm
Diameter (D)	115.6 mm
Fluid Speed ($U_{\infty }$)	12.66 m/s
Helix Angle (δ)	71.45°
Chord Length (C)	15.40 mm
Blade Length	160.08 mm
Hydrofoil Profile	NACA 0015

**Table 4:** Printing parameters of 3D printer.

Parameter	Specification
Fill Density	100%
Speed	100 mm/sec
Layer Height	0.1 mm
Nozzle Temperature	210 °C
Bed Temperature	50 °C
Nozzle Diameter	0.4 mm

**Table 5:** Optimized hybrid renewable energy architectures.

Architecture	PV-H	H	PV
PV (kW)	1.06556	-	8.09370
LA Battery	6	11	17
Hydroelectric (kW)	1.9855	1.9855	-
Converter (kW)	1.4848	2.1875	5.4926
NPC ($)	4902.807	6692.714	18,694.98
COE ($)	0.09418	0.12964	0.35960
Operating Cost ($/yr)	190.2029	336.1491	491.3187
Initial Capital ($)	3411.02	4056.25	14,841.5
PV Capital Cost ($)	1065.561	-	8093.708
PV Output (kWh/yr)	1867.62	-	14,185.92

**Table 6:** Cost details of the individual components.

Component	Capital ($)	Replacement ($)	O&M ($)	Salvage ($)	Total ($)
Hydro-Turbine	600.0	324.56	57	−1.76	1079.66
1 kWh Lead Acid	211	124.85	8	−19.51	363.93
PV System	1065.0	0	3.57	0.00	1148.57
System Converter	445.46	81.38	0	−8.73	518.11

## Data Availability

Not applicable.

## Nomenclature

nZEB	Nearly Zero Energy Building
HRES	Hybrid Renewable Energy Systems
CFD	Computational Fluid Dynamics
ANSYS	Analysis of Systems
HOMER	Hybrid Optimization of Multiple Energy Resources
DNI	Direct Normal Irradiation
IRENA	International Renewable Energy Agency
IEA	International Energy Agency
PV	Photovoltaic
ZEBRA	Zero Energy Building Research Alliance
UK	United Kingdom
USA	United States of America
EMS	Energy Management System
MILP	Mixed-Integer Linear Programming
NASA	National Aeronautics and Space Administration
AC	Alternating Current
DC	Direct Current
O&M	Operation and Maintenance
RMS	Root-Mean-Square
COE	Cost of Energy
NPC	Net Present Cost
BEM	Blade Elemental Momentum
NACA	National Advisory Committee for Aeronautics
GAMBIT	Geometry and Mesh Building Intelligent Tool
3D	Three Dimensional
TKE	Turbulent Kinetic Energy
PLA	Poly-Lactic Acid
PVA	Poly-Vinyl Alcohol
OPEC	Organization of the Petroleum-Exporting Countries
$C_{TANN}$	Total Annual Cost
$E_{ls}$	Energy Delivered to Load
$E_{Grid}$	Energy Delivered to Grid
$CRF_{\left(i,N\right)}$	Capital Recovery Factor
$A_{c\;},\;R$	Cross-Sectional Area of Turbine
i	Interest Rate
ρ	Density of Fluid
$U_{Rel}$	Resultant Velocity
$U_{\infty }$	Velocity of Fluid
α	Angle of Attack
λ	Tip to Speed Ratio
ω	Angular Speed
$C_{Q}$	Torque Coefficient
T	Torque
$C_{p}$	Power Coefficient
$G_{b}$	TKE Due to Buoyancy
$C_{1\varepsilon }$, $C_{2\varepsilon }$	Adjustable Constants
$G_{k}$	TKE Due to Velocity Gradients of Fluid
$S_{k}$ $,\;S_{\varepsilon }$	User-Defined Terms
η	Efficiency
M	Moment of Turbine
q	Dynamic Pressure
$\alpha_{k}$, *α_ε_*	Inverse of the Effective Prandtl Numbers
$Y_{M}$	Ratio of Compressible Turbulence Due to Fluctuating Dilatation to Overall Dissipation Rate
